# Online prenatal trial in mindfulness sleep management (OPTIMISM): protocol for a pilot randomized controlled trial

**DOI:** 10.1186/s40814-020-00675-1

**Published:** 2020-09-14

**Authors:** Ira Kantrowitz-Gordon, Susan M. McCurry, Carol A. Landis, Rachel Lee, Dahee Wi

**Affiliations:** 1 Child, Family, and Population Health Nursing, University of Washington, Box 357262, Seattle, WA 98195 USA; 2 Biobehavioral Nursing and Health Informatics, University of Washington, Box 357266, Seattle, WA 98195 USA

**Keywords:** Mindfulness, Pregnancy, Insomnia, Depression, Internet, Randomized controlled trial, Study protocol

## Abstract

**Background:**

Sleep deficiency affects a majority of pregnant women with significant impact on daily function, mood, and pregnancy and birth outcomes. This ongoing study combines two evidence-based strategies for improving sleep and mood, mindfulness meditation and cognitive-behavioral therapy for insomnia (CBT-I), in a unique online format to address the particular needs of pregnant women. The purpose of this study is to test the feasibility and estimate the efficacy of this novel 6-week online mindfulness meditation intervention to help pregnant women in remission from depression self-manage insomnia.

**Methods:**

This is a two-arm, parallel group randomized controlled trial. A total of 50 pregnant women between 12 and 28 weeks gestation will be recruited from the community and randomly assigned to a mindfulness or education-only control group in a 1:1 ratio. During the study, all participants will complete six weekly online modules, daily sleep diaries, and optional participation in a treatment-specific online discussion forum. Feasibility outcome measures will include study recruitment, retention, intervention adherence (number of online modules completed, number of meditation days per week), and intervention acceptability (8-item questionnaire). The primary clinical outcome measure will be sleep quality measured with the Pittsburgh Sleep Quality Index. Secondary outcome measures will include sleep measured with actigraphy and diaries (sleep efficiency, total sleep time, total wake time), Patient-Reported Outcomes Measurement Information System (PROMIS) measures (fatigue, sleep-related impairment, sleep disturbance); mood (depression, anxiety, positive affect, quality of life); and self-management and behavior change (potential self-efficacy, self-regulation, sleep problem acceptance, and trait mindfulness). Assessments will occur at baseline and post-intervention; an additional acceptability survey will be completed 4 weeks postpartum. Analyses will examine within-group differences in outcome change scores from baseline to post-intervention. Open-ended feedback will be analyzed using qualitative content analysis.

**Discussion:**

This research is innovative in addressing sleep in pregnancy using a self-management research design and methods that can be accessible and cost-effective for large numbers of pregnant women. The results from this study will inform intervention refinement and efficacy testing of the intervention in a larger randomized controlled trial.

**Trial registration:**

ClinicalTrials.gov, NCT04016428. Registered on 11 July 2019. Updated version registered on 26 July 2019.

## Background

Poor sleep quality is experienced by 76% of women during pregnancy [1]. Sleep deficiency, including an inadequate amount of sleep and poor sleep quality, has been associated with poor maternal and fetal outcomes, such as gestational diabetes [2], preterm birth [3], and cesarean delivery [4]. Poor sleep is also associated with antenatal and postpartum depression symptoms [5], with long-term consequences for maternal and child well-being [6]. A majority of pregnant women prefer non-pharmacological treatments for symptoms like insomnia and depression, possibly because of concerns about fetal or neonatal health during pregnancy and lactation [7, 8]. Preliminary evidence suggests that behavioral approaches such as cognitive behavioral therapy for insomnia (CBT-I) are efficacious in pregnancy [9, 10].

### Mindfulness-based interventions in pregnancy

Mindfulness is a behavioral self-management strategy well suited as an adjunct with CTB-I for treatment of sleep deficiency. Mindfulness-based stress reduction (MBSR) is a non-pharmacological treatment approach that teaches the ability to pay attention in the moment without judgment through daily meditation practice [11]. MBSR and similar mindfulness-based interventions (MBIs) have been shown efficacious at reducing depressive and anxiety symptoms in pregnancy and postpartum, but sleep quality was not a key outcome in these studies [12, 13]. Mindfulness approaches raise awareness of reactive tendencies, such as self-judgment [14], which can promote arousal during active efforts to manage sleep and depression. The theoretical basis for mindfulness as a self-management strategy for sleep and depression is that principles of mindfulness (acceptance, awareness, and nonjudgment) can reduce sleep-related arousal and rumination that are common both to insomnia and depression [15]. An awareness of sleepiness that is cultivated by mindfulness approaches can also be integrated into strategies to increase sleep efficiency by going to bed and staying in bed only during a state of sleepiness [16]. Mindfulness interventions have been correlated with components of self-management, including self-efficacy [17] and intrinsic motivation [18], and may correlate with increased patient activation [19]. Limited research suggests that MBIs can be effective in treating insomnia [16] although such interventions have not been tested in pregnancy. Given the demonstrated benefit of mindfulness training in pregnancy for improving depression symptoms and preventing depression relapse [12, 20], mindfulness may be especially suited, in combination with CBT-I, for treating insomnia in pregnant women with a history of depression.

The online prenatal trial in mindfulness sleep management (OPTIMISM) intervention combines elements of two mindfulness interventions, mindfulness-based childbirth and parenting (MBCP) [21] and mindfulness-based therapy for insomnia (MBTI) [16]. Delivery of content adapted for the context of pregnancy has the potential to make the program more relevant, accessible, and cost-effective. There is accumulating evidence that mindfulness-based interventions can be delivered online with similar efficacy as in-person classes on reducing stress, depression, and anxiety [22], but this approach has not been tested for improvement of sleep outcomes in pregnant women. The current pilot randomized controlled trial builds on previous research to investigate whether OPTIMISM positively impacts sleep, depression symptoms, and self-management variables. Pregnant women with a history of depression are targeted for this study because of the correlation between sleep disturbance and depression symptoms.

### Five hypotheses

The primary hypotheses for the trial are as follows:

1. The OPTIMISM intervention will be feasible and acceptable to participants.
2. The OPTIMISM intervention will be associated with improved global sleep quality by the end of treatment (6 weeks) compared to education-only control (EOC).

The secondary hypotheses are that, compared to EOC:

3. The OPTIMISM intervention will be associated with post-treatment (6 week) reductions in fatigue, sleep-related impairment, and sleep disturbance.
4. The OPTIMISM intervention will be associated with post-treatment improvements in depression and anxiety symptoms, positive affect, and health status.

The exploratory hypothesis for the trial is that, compared to EOC:

5. The OPTIMISM intervention will be associated post-treatment with increased self-efficacy, patient activation, motivation, and mindfulness practice.

## Methods

### Study design

The study will use a randomized controlled pretest-posttest design to describe the feasibility and acceptability of the OPTIMISM intervention compared to an active control to estimate effect size. Data will be collected at baseline (T1) and post-intervention (T2), and postpartum (T3). This protocol was registered on 11 July 2019 (NCT04016428) with ClinicalTrials.gov, https://clinicaltrials.gov/ct2/show/NCT04016428. Any trial amendments will be approved by the Institutional Review Board before implementation and will be reported to the trial registry. Table 1 shows a flowchart of the study and Additional file 1 provides the Standard Protocol Items: Recommendations for Interventional Trials (SPIRIT) checklist.

**Table 1:**
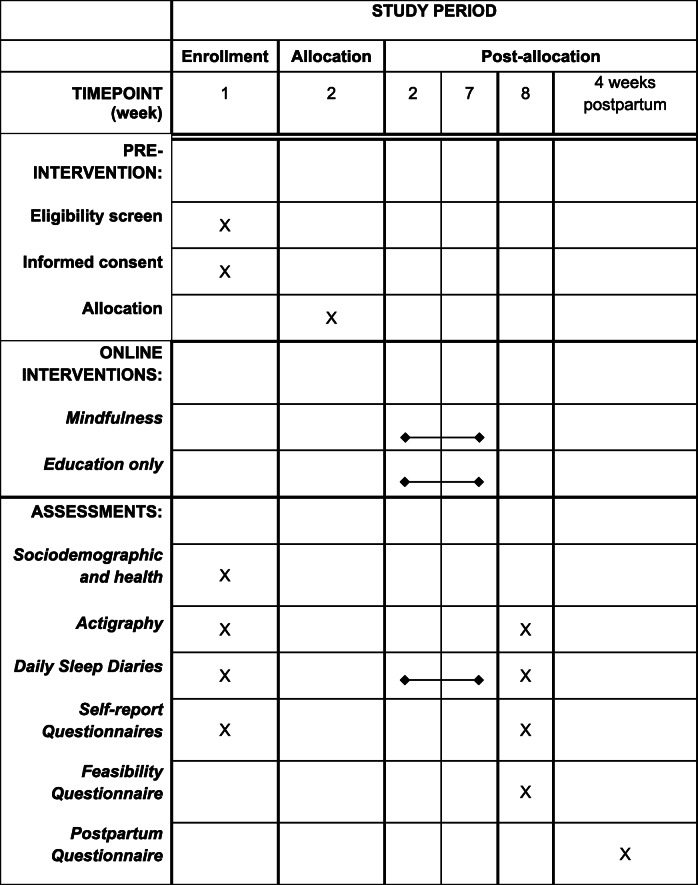
SPIRIT schedule of enrollment, interventions, and assessment

### Participants

We plan to recruit 50 pregnant women with self-identified poor sleep and history of depression. Power analysis was performed for the primary outcome, sleep quality as measured by the PSQI. Small open pilot studies of cognitive behavioral therapy for insomnia in pregnancy [9] and postpartum [23] have found large effect sizes, Cohen’s *d* of 1.7, for improvement in the PSQI. We powered our study to detect a smaller effect size because effect size estimates from the published trials could be unreliable due to small sample sizes. We estimated the least detectable difference for sleep quality between the two groups at post-test based on a 2-sided *t* test of means (*α* = .05). The proposed sample size of 50 would leave 22 women per group (assuming 12% attrition) to provide outcome data and yield 80% power for a smallest detectable effect size of .87 (G*Power 3.1.9.4) [24].

Recruitment flyers will be distributed to community-based prenatal care clinics, high-risk pregnancy clinics, maternity service providers, and perinatal mental health providers in the Seattle metropolitan area, USA. Posts will be made to social media accounts, Facebook, Nextdoor, and Meetup groups. The study will be posted on research recruitment sites such as Participate in Research. Posts guide potential participants to the study webpage, phone number, and email. Potential participants will also be identified through an electronic medical record screening and sent an email with information about the study.

### Inclusion and exclusion criteria

Eligible participants will be women who are at least 18 years old with a viable pregnancy between 12–28 weeks gestation, with self-reported insomnia (score > 7 on the Insomnia Severity Index) and a history of depression currently in remission (score < 3 on the Patient Health Questionnaire-2). Participants need to have English fluency and access to an Internet-enabled smartphone, tablet, or computer. Exclusion criteria are known severe congenital fetal anomalies, fetal demise, or expected neonatal death; other significant psychiatric illness requiring treatment; current hospitalization; prior diagnosis of obstructive sleep apnea or restless leg syndrome; positive self-report screen for restless leg syndrome; regular mindfulness or meditative practice (at least weekly); and regular night-shift work. Participation will be allowed regardless of whether or not the potential participant is currently receiving psychotropic medications or psychotherapy.

### Screening, consent, and enrollment

Interested potential participants will be screened by telephone or online using a questionnaire to determine whether they meet inclusion and exclusion criteria. Potential participants will be notified of their eligibility immediately when screening is completed by phone; if screened online, they will be notified by telephone within a few days. Eligible and interested participants will be scheduled for the enrollment visit with a research assistant at a mutually agreeable location to discuss the study, answer questions, and provide oral and written informed consent. Participants will receive a paper copy of the signed consent.

### Baseline assessment

Baseline assessment will include completion of self-reported questionnaires (demographics, pregnancy and general health status, subjective sleep, mood, and self-management variables) and sleep monitoring with actigraphy and sleep diaries. Verbal and written instructions will be provided at the enrollment visit for wearing an actigraph on the non-dominant wrist for 8 consecutive days (7 nights), and participants will commence daily online sleep diaries. A link to the online questionnaires and sleep diaries will be sent to participants by email after the first visit. After all baseline measures are completed, a research team member will meet with participants to retrieve the actigraph, notify them of their randomization to receive the OPTIMISM or active control condition, and provide instruction in completing the online modules. A detailed description of all measures is presented below.

### OPTIMISM intervention

The OPTIMISM intervention consists of six weekly online self-directed learning modules about mindfulness meditation, sleep challenges in pregnancy, and behavioral strategies to improve sleep. This program adapts elements of two in-person mindfulness-based interventions: mindfulness-based childbirth and parenting (MBCP) [21] and mindfulness-based therapy for insomnia (MBTI) [16]. The emphasis of intervention activities will be the use of mindful awareness as a sleep self-management technique to increase total sleep time (TST) and sleep efficiency (SE). The intervention also incorporates elements of sleep hygiene, sleep restriction, and stimulus control. Sleep restriction is a strategy to restrict the time in bed to avoid prolonged middle of the night awakenings while not reducing total sleep time [25]. Stimulus control is a strategy to avoid arousal while in bed by only going to bed when sleepy and getting out of bed when unable to fall back asleep in the middle of the night [26]. Intervention modules were developed using Articulate Storyline, Version 3.31, and published online through Articulate Online (Articulate Global, Inc.). Each weekly module contains didactic content on sleep, pregnancy, and mindfulness meditation using interactive text and video, and audio meditations (Table 2). The duration of each module is approximately 20 min and can be completed at a time chosen by the participant. There is also a discussion board for interaction with other participants in the OPTIMISM arm and research staff. Support during daytime hours will be available by text message, email, or phone communication with research staff. Reminders to complete daily sleep diaries and weekly modules will be sent by text or email at intervals agreeable to participants. Every morning during the course, participants will complete an online or paper sleep diary, modified to include pregnancy symptoms interfering with sleep, along with reports of daily meditation practice. Weekly feedback on adherence to the participant’s individualized recommended sleep schedule will be provided to participants by emailed report beginning with the end of the second week of the program. The feedback will include a visual representation of their diary-reported bed time and wake time superimposed over their recommended sleep schedule along with any recommended changes to their schedule for the coming week.

**Table 2:** Session content for mindfulness arm

Module	Content	Mindfulness practices
1. Introduction	● Facts about sleep ● Sleep and health ● Sleep in pregnancy ● Pregnancy sleep strategies ● Introduction to mindfulness	● Mindfully eating a raisin ● Awareness of breathing
2. Timing	● Diet and sleep ● Insomnia ● Sleep schedule and window ● Bedtime routines ● Mindfulness and sleep ● Mindfulness and labor	● Body scan
3. Activity	● Reviewing and adjusting sleep schedule ● Stimulus control ● Mindful movement ● Physical activity in pregnancy ● Movement and rest in labor	● Mindful walking
4. Acceptance	● Reviewing and adjusting sleep schedule ● Choices and control in labor ● Acceptance and letting go	● Being with baby
5. Self-compassion	● Reviewing and adjusting sleep schedule ● Mood and sleep ● Self-care and self-compassion	● Loving kindness
6. Postpartum and beyond	● Reviewing and adjusting sleep schedule ● Physical recovery postpartum ● Newborn sleep ● Postpartum sleep ● Postpartum mindfulness	● Being with baby

### Control intervention

The education-only control (EOC) intervention will deliver weekly online modules on sleep, pregnancy, and childbirth developed on Articulate Storyline and published on Articulate Online (Table 3). The duration of each module is approximately 20 min and can be completed at a time chosen by the participant. An active control condition instead of treatment as usual was chosen to control for non-specific attention and self-monitoring effects [27]. This will allow any outcome differences between the two groups to be attributable to the active treatment components of the OPTIMISM intervention: mindful awareness, sleep hygiene education, stimulus control, and sleep restriction. The EOC condition will not include any mindfulness content or meditations, sleep scheduling/bed restriction, or stimulus control recommendations. EOC participants will complete daily sleep diaries and have access to an online discussion board limited to EOC group participants and research staff.

**Table 3:** Session content for education-only arm

Module	Content
1. Introduction to sleep	● Facts about sleep ● Sleep and health ● Sleep in pregnancy
2. Sleep strategies	● Diet and sleep ● Sleep and environment ● Bedtime routines ● Pregnancy sleep strategies
3. Physical activity	● Benefits of physical activity in pregnancy ● How to exercise in pregnancy ● Positions, activity, and rest in labor
4. Mood and sleep	● Poor sleep, emotions, and mood ● Depression in pregnancy ● Self-care
5. Medications and sleep	● Over the counter medications ● Prescription medications ● Substances (caffeine, alcohol, nicotine) ● Labor pain medications
6. Sleep postpartum	● Physical recovery postpartum ● Newborn sleep ● Postpartum sleep ● Physical activity postpartum

### Methods to protect against sources of bias

#### Randomization and allocation

After completion of the baseline assessment, eligible women will be block-randomized to receive OPTIMISM or active control. A computer-generated randomization allocation sequence uploaded to the study data management platform will automatically assign participants to a treatment condition immediately prior to orientation to the online intervention platform by a research assistant. Participants will be enrolled in the online interventions on a continuous basis because there is no need for a cohort of participants as there would be in an in-person group intervention.

#### Blinding

Participants will know that the research study is testing two alternative online interventions to improve sleep in pregnancy but will not be informed of the nature of the two interventions. This reduces the likelihood of EOC participants seeking out mindfulness treatments independently, or dropping prematurely because of disappointment over being assigned to a control group [27]. Online self-report measures will be scored automatically.

#### Simultaneous interventions

Participation in this study will not preclude adjunctive treatments from prenatal care providers, such as referral to a sleep specialist, medications, or behavioral treatments including mindfulness training. Information about any adjunctive sleep treatments will be collected at the post-intervention assessment to control for this variable in analyses. Use of sleep medications will also be collected in the daily sleep diaries.

### Schedule of visits

Study participants will complete self-report measures and actigraphy at study entry (T1) and 6-weeks later after intervention completion (T2). Participants will complete sleep diaries from study entry through completion of the second actigraphy data collection week at T2. An additional brief self-report assessment will occur approximately 4 weeks postpartum to collect data on birth outcomes and whether participants have continued to use intervention strategies during the postpartum period. Participants will receive a $50 retail gift card upon completion of each of the T1/T2 assessments in the study.

Research staff will monitor the online platform weekly for module completion. Research staff will communicate with participants if they fail to complete weekly modules or sleep diaries and will help with any technical difficulties accessing the online platforms. If a participant discontinues participation in their assigned intervention without completion, they will be encouraged to complete the post-assessment and will be included in intention-to-treat outcome analyses.

### Outcome measures

#### Feasibility

The feasibility of the study involves examination of recruitment, retention, intervention adherence, and acceptability. The feasibility of recruitment will be determined by calculating the proportion of enrolled participants from those screened for eligibility from the varied recruitment sources. Retention will be measured by the percentage of participants completing the study through the post-assessment. Intervention adherence will be measured as the number of OPTIMISM or EOC online modules completed, with a range of 0 to 6. Meditation adherence for OPTIMISM participants will be measured by the average number of days per week the participant practiced at least one meditation, with a range of 0 to 7. Use of specific meditation practices will be measured in the OPTIMISM sleep diaries and automatically tracked by the intervention website. Intervention acceptability will be measured by an investigator-developed self-report 8-item questionnaire adapted from a similar feasibility trial of a mindfulness program [28]. Questions address intervention satisfaction, usefulness for managing sleep and stress, and ease of use. Each item rates acceptability on a 5-point Likert-type scale ranging from 1 (strongly disagree) to 5 (strongly agree). The range of total scores is 8 to 40, with higher scores indicating increased acceptability. Additional questions will address the acceptability of the daily sleep diaries, online modules, and mindfulness meditations. Open-ended qualitative questions will solicit general feedback on the intervention and aspects that were found to be helpful and unhelpful. Acceptability questions will focus both on the content of the intervention as well as process (Internet delivery, mobile device compatibility, and time involved).

#### Primary clinical outcome

The primary clinical outcome will be sleep quality, measured with *the Pittsburgh Sleep Quality Index (PSQI),* a 19-item self-rating of overall sleep quality and usual sleep habits over the previous month [29]. Total scores range from 0 to 21, with higher scores indicating worse sleep quality. Survey items are rated on a 0-3 Likert-type scale. A PSQI global score > 5 is sensitive and specific for distinguishing good and poor sleepers [29]. A change of 3.0 is considered a clinically important difference in the PSQI score [30]. A lower PSQI score indicates improved sleep quality and sleep habits.

#### Secondary outcomes

Secondary outcomes include objective and subjective sleep parameters, mood, well-being, and quality of life. The inclusion of these measures met requirements of the funder to utilize common data elements for inclusion of study data in the Common Data Repository for Nursing Science (https://cdrns.nih.gov/). These secondary outcome measures will inform choice of instruments for subsequent clinical trials.

*Objective and subjective sleep* measures will be assessed with wrist actigraphy (Actiwatch Spectrum Plus, Philips Respironics, Bend, OR) and online or paper sleep diaries for 8 days at baseline and post-intervention. *Sleep efficiency* will be calculated from total sleep time divided by time in bed. Increased sleep efficiency is a better outcome. *Total wake time* will include all time spent awake during the rest period, measured in minutes. This will be calculated as the sum of sleep onset latency (time from getting into bed to falling asleep) and wake after sleep onset (time awake after sleep onset). *Total sleep time* is the sum of all time spent asleep, measured in minutes. This will be calculated as the difference between time in bed and total wake time.

*Patient-Reported Outcomes Measurement Information System (PROMIS) Sleep Disturbance Short Form (v.6a)* is a six-item self-report questionnaire that measures perception of the quality of sleep and difficulty falling asleep over the past 7 days [31]. Items are measured on a 5-point Likert-type scale. Total scores range from 6 to 30, with higher scores indicating more disturbed sleep.

*PROMIS Sleep-Related Impairment Short Form (v.8a)* is an eight-item self-report questionnaire for perceptions of alertness, sleepiness, and tiredness during usual waking hours, and perceived functional impairments during wakefulness associated with sleep problems or impaired alertness [31]. Items are measured on a 5-point Likert-type scale ranging from 1 (not at all) to 5 (very much). Total scores range from 8 to 40, with higher scores indicating increased impairment over the past 7 days.

*PROMIS Fatigue Short Form (v.6a)* is a six-item self-report questionnaire for reporting subjective feelings of tiredness and the impact on ability to function normally over the past 7 days [32]. Items are scored on a 5-point Likert-type scale. Total scores range from 6 to 30, with higher scores indicating increased fatigue.

*PROMIS Anxiety Short Form (v.6a)* is a six-item self-report questionnaire for anxiety symptoms in the past week [33]. Total scores range from 6 to 30, with higher scores indicating increased symptoms of anxiety. Items are scored on a 5-point Likert-type scale ranging from 1 (never) to 5 (always).

*PROMIS Depression Short Form (v.6a)* is a six-item self-report questionnaire for depression symptoms in the past week [33]. Total scores range from 6 to 30, with higher scores indicating increased symptoms of depression.

*Edinburgh Postnatal Depression Scale (EPDS)* [34] is a ten-item self-report questionnaire for depression symptoms in the past week validated for use during pregnancy and postpartum [35]. The EPDS does not measure somatic symptoms of depression, which can be normal in pregnancy or postpartum. Items are scored from 0 to 3, and the scale score is the sum of all ten items. Total scores range from 0 to 30, with higher scores indicating increased symptoms of depression. Total scores ≥ 12 indicate increased risk for depression, which must then be clinically validated [36]. The EPDS has adequate internal consistency with Cronbach’s alpha .87 [34].

*Neuro-QOL Positive Affect and Well-Being Short Form* is a nine-item self-report questionnaire for recent frequency of positive emotions [37]. Each item rates frequency on a 5-point Likert-type scale ranging from 1 (never) to 5 (always).Total scores range from 9 to 45, with higher scores indicating increased positive emotions.

*Short Form (36) Health Survey (SF-36)* is a 36-item, patient-reported survey of patient health status and quality of life [38]. The SF-36 is a widely used indicator of overall health status across vitality, physical functioning, bodily pain, general health perceptions, physical role functioning, emotional role functioning, social role functioning, and mental health. Total scores range from 0 to 100, with lower scores indicating increased disability and higher scores indicating higher health and quality of life.

#### Exploratory outcomes

Exploratory outcomes include self-efficacy, self-regulation, acceptance, and state mindfulness. These outcomes are conceptually related to the proposed mechanism of the intervention for improving sleep.

*PROMIS Self-Efficacy for Managing Emotions*—Short Form (v.4a) is a four-item self-report questionnaire for measuring confidence in handling emotions [39]. Total scores range from 4 to 20, with higher scores indicating greater ability to handle emotions.

*Index of Self-Regulation (ISR)* is a nine-item self-report questionnaire designed to measure individuals’ level of motivation and self-regulation for health-related behavior change [40]. Total scores range from 6 to 54, with higher scores indicating greater self-regulation.

*Sleep Problem Acceptance Questionnaire (SPAQ)* is an 8-item self-report questionnaire that assesses acceptance of insomnia [41]. Total scores range from 0 to 48, with higher scores indicating a higher level of acceptance. Increased acceptance of insomnia is believed to be a mechanism for reducing the struggle to fall asleep [41].

*Five Facet Mindfulness Questionnaire Short Form (FFMQ-SF)* is a 24-item self-report questionnaire that measures the trait-like tendency to be mindful in daily life [42]. It includes five subscales, measuring different aspects of mindfulness observing, describing, acting with awareness, nonjudging, and nonreactivity. Items are scored on a 5-point Likert-type scale ranging from 1 (never or very rarely true) to 5 (very often or always true). Four of the subscale scores range from 5 to 25; one subscale “observing” has a range of 4 to 20. Higher scores indicate increased mindfulness. The FFMQ-SF has been validated in pregnancy, and Cronbach’s alpha = .86 [43].

#### Baseline characteristics

Demographic and health status information will be collected by self-report at baseline. Demographic information includes age, gender, race, ethnicity, education level, employment status, marital status, and number of people in household. Pregnancy status includes parity, gestational age, fetal number, height, current weight, pre-pregnancy weight, and pregnancy complications. Health information includes chronic medical and psychiatric conditions, lifetime use of mental health services, current medications, substance use, and disabilities.

### Adverse events

There are no known risks to mindfulness meditation in pregnancy. If any adverse events occur, such as identified increased risk of depression or suicidality, the principal investigator will offer community resources (help and referral lines, therapists and psychiatric providers, and support groups) and encourage the participant to contact her primary care provider or prenatal care provider for appropriate management and follow-up. Adverse events will be reported to a Data Safety Monitoring Board composed of experienced clinical researchers and clinicians and to the University of Washington Institutional Review Board.

### Data analyses

PROMIS measures will be scored using the HealthMeasures Scoring Service (www.healthmeasures.net). Descriptive statistics will be calculated on demographics, pregnancy characteristics, and primary and secondary outcome variables. All data will be examined for normality and outliers. Non-normal variables will be corrected by an appropriate transformation. Analysis of primary and secondary outcomes will be conducted based on group allocation (intention-to-treat). The percentage and patterns of missing data from all study measures will be calculated, examined, and handled case wise. We will explore group changes with time by including the interaction between time and group. We will compare predicted mean scores and confidence intervals to perform the specific comparisons of interest between the OPTIMISM treatment and control groups. We will report effect sizes (Eta squared) to inform future clinical trials. The means and distributions of acceptability questions will be examined for each intervention separately. The threshold for acceptability will be a score of 4 or 5 (agree or strongly agree) for the majority of participants.

Open-ended acceptability questions will be analyzed separately for each intervention using qualitative content analysis [44]. Responses will be downloaded into a single Word file and coded independently by two investigators. Response units will be coded and then organized into categories and themes. The investigators will meet to discuss coding and resolve differences in data analysis. The analysis will facilitate identification of intervention strengths and areas for improvement to inform revisions of the intervention for future clinical trials.

Study data will be collected and managed using Research Electronic Data Capture (REDCap) tools [45] hosted at the Institute of Translational Health Sciences (ITHS). REDCap is a secure, web-based application designed to support data capture for research studies, providing (1) an intuitive interface for validated data entry; (2) audit trails for tracking data manipulation and export procedures; (3) automated export procedures for seamless data downloads to common statistical packages; and (4) procedures for importing data from external sources. Participant identifiers will be kept separately from participant data in a locked office or secure electronic files.

## Discussion

Despite the high prevalence of sleep disturbance during pregnancy, effective behavioral treatments are largely inaccessible because of time, cost, and insufficient numbers of trained therapists [46]. The goal of this study is to determine whether an online intervention can potentially address this gap in care. Online delivery has many advantages, including automation, scalability, and cost-effectiveness. To our knowledge this is the first online mindfulness intervention to address sleep deficiency in pregnancy.

Limitations of this study include the possibility that participants in either group may seek other treatments for insomnia outside of their assigned treatment, which may limit the ability to detect differences between the two treatments. To control for engagement in alternative treatments, we ask participants about these potential exposures during the post-intervention assessment. Use of medications affecting sleep is also accounted in the daily sleep diaries. Another limitation may come from the lack of connection with others who have insomnia in pregnancy that may develop in an in-person group intervention. This study uses an online discussion board as a surrogate for developing a sense of social connection with others with insomnia. The discussion board is prepopulated with content to make participation more welcoming. Strengths of this study include provision of an active control treatment based on sleep education [27] tailored to pregnancy. Use of daily sleep diaries in both treatments controls for any effect from self-monitoring that is separate from the treatment approach. An ongoing question in mindfulness science is the optimal “dose” of mindfulness treatment, including the number of class sessions and the amount of mindfulness practice [47, 48]. We will track completion of the online modules as well as number and type of mindfulness practices through the sleep diary and automatic tracking of access to the audio meditations on the online intervention platform.

The results from this trial will help determine the feasibility, acceptability, and preliminary efficacy of an online mindfulness sleep intervention for pregnant women with insomnia. Results will inform refinement of the intervention content and mode of delivery as well as use of daily online sleep diaries specific for pregnancy. This will prepare us for larger efficacy trials in pregnant women with insomnia and at risk for depression.

### Trial status

This study is recruiting participants. We expect to have completed enrollment by December 2020.

## Supplementary information

**Additional file 1.** Includes a copy of the SPIRIT checklist.

## Data Availability

There are no plans for interim analyses or interim data sharing. Data will not be released before trial completion and will be analyzed independently by the study team. Data will be shared via the Common Data Repository for Nursing Science (cdRNS) at the National Institute of Nursing Research (https://cdrns.nih.gov/).

## Abbreviations

CBT-I: Cognitive Behavioral Therapy for Insomnia
EPDS: Edinburgh Postnatal Depression Scale
EOC: Education-only control
FFMQ-SF: Five Factor Mindfulness Questionnaire Short Form
ISR: Index of Self-Regulation
MBCP: Mindfulness-based childbirth and parenting
MBSR: Mindfulness-based stress reduction
MBTI: Mindfulness-based treatment of insomnia
OPTIMISM: Online prenatal trial in mindfulness sleep management
PHQ-9: Patient Health Questionnaire
PROMIS: Patient-Reported Outcomes Measurement Information System
SF-36: Short Form Health Survey
SPAQ: Sleep Problem Acceptance Questionnaire
SE: Sleep efficiency
TST: Total sleep time

## References

[1] Mindell JA, Cook RA, Nikolovski J (2015). Sleep patterns and sleep disturbances across pregnancy. Sleep Med.

[2] Rawal S, Hinkle SN, Zhu Y, Albert PS, Zhang C (2017). A longitudinal study of sleep duration in pregnancy and subsequent risk of gestational diabetes: findings from a prospective, multiracial cohort. Am J Obstet Gynecol.

[3] Felder JN, Baer RJ, Rand L, Jelliffe-Pawlowski LL, Prather AA (2017). Sleep disorder diagnosis during pregnancy and risk of preterm birth. Obstet Gynecol.

[4] Palagini L, Gemignani A, Banti S, Manconi M, Mauri M, Riemann D (2014). Chronic sleep loss during pregnancy as a determinant of stress: impact on pregnancy outcome. Sleep Med.

[5] Tomfohr LM, Buliga E, Letourneau NL, Campbell TS, Giesbrecht GF (2015). Trajectories of sleep quality and associations with mood during the perinatal period. Sleep..

[6] Slomian J, Honvo G, Emonts P, Reginster JY, Bruyère O (2019). Consequences of maternal postpartum depression: a systematic review of maternal and infant outcomes. Womens Health (Lond).

[7] Dimidjian S, Goodman SH (2014). Preferences and attitudes toward approaches to depression relapse/recurrence prevention among pregnant women. Behav Res Ther.

[8] Sedov ID, Goodman SH, Tomfohr-Madsen LM (2017). Insomnia treatment preferences during pregnancy. J Obstet Gynecol Neonatal Nurs.

[9] Tomfohr-Madsen LM, Clayborne ZM, Rouleau CR, Campbell TS (2017). Sleeping for two: an open-pilot study of cognitive behavioral therapy for insomnia in pregnancy. Behav Sleep Med.

[10] Manber R, Bei B, Simpson N, Asarnow L, Rangel E, Sit A (2019). Cognitive behavioral therapy for prenatal insomnia: a randomized controlled trial. Obstet Gynecol.

[11] Kabat-Zinn J (1982). An outpatient program in behavioral medicine for chronic pain patients based on the practice of mindfulness meditation: theoretical considerations and preliminary results. Gen Hosp Psychiatry.

[12] Dimidjian S, Goodman SH, Felder JN, Gallop R, Brown AP, Beck A (2016). Staying well during pregnancy and the postpartum: a pilot randomized trial of mindfulness-based cognitive therapy for the prevention of depressive relapse/recurrence. J Consult Clin Psychol.

[13] Zemestani M, Fazeli NZ (2019). Effectiveness of mindfulness-based cognitive therapy for comorbid depression and anxiety in pregnancy: a randomized controlled trial. Arch Womens Ment Health.

[14] Ong JC, Ulmer CS, Manber R (2012). Improving sleep with mindfulness and acceptance: a metacognitive model of insomnia. Behav Res Ther.

[15] Ong JC, Moore C (2019). What do we really know about mindfulness and sleep health?. Curr Opin Psychol.

[16] Ong JC, Manber R, Segal Z, Xia Y, Shapiro S, Wyatt JK (2014). A randomized controlled trial of mindfulness meditation for chronic insomnia. Sleep..

[17] Turner JA, Anderson ML, Balderson BH, Cook AJ, Sherman KJ, Cherkin DC (2016). Mindfulness-based stress reduction and cognitive behavioral therapy for chronic low back pain: similar effects on mindfulness, catastrophizing, self-efficacy, and acceptance in a randomized controlled trial. Pain..

[18] Ruffault A, Bernier M, Juge N, Fournier JF (2016). Mindfulness may moderate the relationship between intrinsic motivation and physical activity: a cross-sectional study. Mindfulness..

[19] Magnezi R, Glasser S, Shalev H, Sheiber A, Reuveni H (2014). Patient activation, depression and quality of life. Patient Educ Couns.

[20] Duncan LG, Cohn MA, Chao MT, Cook JG, Riccobono J, Bardacke N (2017). Benefits of preparing for childbirth with mindfulness training: a randomized controlled trial with active comparison. BMC Pregnancy Childbirth.

[21] Duncan LG, Bardacke N (2010). Mindfulness-based childbirth and parenting education: promoting family mindfulness during the perinatal period. J Child Fam Stud.

[22] Fish J, Brimson J, Lynch S (2016). Mindfulness interventions delivered by technology without facilitator involvement: what research exists and what are the clinical outcomes?. Mindfulness (N Y).

[23] Swanson LM, Flynn H, Adams-Mundy JD, Armitage R, Arnedt JT (2013). An open pilot of cognitive-behavioral therapy for insomnia in women with postpartum depression. Behav Sleep Med.

[24] Faul F, Erdfelder E, Lang AG, Buchner A (2007). G*power 3: a flexible statistical power analysis program for the social, behavioral, and biomedical sciences. Behav Res Methods.

[25] Spielman AJ, Saskin P, Thorpy MJ (1987). Treatment of chronic insomnia by restriction of time in bed. Sleep..

[26] Bootzin RR, Koocher GP, Norcross JC, Greene BA (2013). Implementing stimulus control therapy for insomnia. Psychologists’ desk reference.

[27] Balderson BH, McCurry SM, Vitiello MV, Shortreed SM, Rybarczyk BD, Keefe FJ (2016). Information without implementation: a practical example for developing a best practice education control group. Behav Sleep Med.

[28] Price C, Kantrowitz-Gordon I, Calhoun R (2019). A pilot feasibility study of mindfulness childbirth education for women with a history of sexual trauma. Complement Ther Clin Pract.

[29] Buysse DJ, Reynolds CF, Monk TH, Berman SR, Kupfer DJ (1989). The Pittsburgh sleep quality index: a new instrument for psychiatric practice and research. Psychiatry Res.

[30] Hughes CM, McCullough CA, Bradbury I, Boyde C, Hume D, Yuan J (2009). Acupuncture and reflexology for insomnia: a feasibility study. Acupunct Med.

[31] Buysse DJ, Yu L, Moul DE, Germain A, Stover A, Dodds NE (2010). Development and validation of patient-reported outcome measures for sleep disturbance and sleep-related impairments. Sleep..

[32] Cella D, Riley W, Stone A, Rothrock N, Reeve B, Yount S (2010). The patient-reported outcomes measurement information system (PROMIS) developed and tested its first wave of adult self-reported health outcome item banks: 2005-2008. J Clin Epidemiol.

[33] Pilkonis PA, Choi SW, Reise SP, Stover AM, Riley WT, Cella D (2011). Item banks for measuring emotional distress from the patient-reported outcomes measurement information system (PROMIS®): depression, anxiety, and anger. Assessment..

[34] Cox JL, Holden JM, Sagovsky R (1987). Detection of postnatal depression. Development of the 10-item Edinburgh postnatal depression scale. Br J Psychiatry.

[35] Kozinszky Z, Dudas RB (2015). Validation studies of the Edinburgh postnatal depression scale for the antenatal period. J Affect Disord.

[36] Murray D, Cox JL (1990). Screening for depression during pregnancy with the Edinburgh depression scale (EPDS). J Reprod Infant Psychol.

[37] Salsman JM, Victorson D, Choi SW, Peterman AH, Heinemann AW, Nowinski C (2013). Development and validation of the positive affect and well-being scale for the neurology quality of life (Neuro-QOL) measurement system. Qual Life Res.

[38] McHorney CA, Ware JE, Lu JF, Sherbourne CD (1994). The MOS 36-item short-form health survey (SF-36): III. Tests of data quality, scaling assumptions, and reliability across diverse patient groups. Med Care.

[39] Gruber-Baldini AL, Velozo C, Romero S, Shulman LM (2017). Validation of the PROMIS(®) measures of self-efficacy for managing chronic conditions. Qual Life Res.

[40] Fleury J (1998). The index of self-regulation: development and psychometric analysis. J Nurs Meas.

[41] Bothelius K, Jernelöv S, Fredrikson M, McCracken LM, Kaldo V (2015). Measuring acceptance of sleep difficulties: the development of the sleep problem acceptance questionnaire. Sleep..

[42] Bohlmeijer E, ten Klooster PM, Fledderus M, Veehof M, Baer R (2011). Psychometric properties of the five facet mindfulness questionnaire in depressed adults and development of a short form. Assessment..

[43] Kantrowitz-Gordon I (2017). Factor structure and external validity of the five facet mindfulness questionnaire in pregnancy. Mindfulness..

[44] Graneheim UH, Lundman B (2004). Qualitative content analysis in nursing research: concepts, procedures and measures to achieve trustworthiness. Nurse Educ Today.

[45] Harris PA, Taylor R, Thielke R, Payne J, Gonzalez N, Conde JG (2009). Research electronic data capture (REDCap)--a metadata-driven methodology and workflow process for providing translational research informatics support. J Biomed Inform.

[46] Koffel E, Bramoweth AD, Ulmer CS (2018). Increasing access to and utilization of cognitive behavioral therapy for insomnia (CBT-I): a narrative review. J Gen Intern Med.

[47] Greenberg J, Braun TD, Schneider ML, Finkelstein-Fox L, Conboy LA, Schifano ED (2018). Is less more? A randomized comparison of home practice time in a mind-body program. Behav Res Ther.

[48] Carmody J, Baer RA (2009). How long does a mindfulness-based stress reduction program need to be? A review of class contact hours and effect sizes for psychological distress. J Clin Psychol.

